# Practical Notes and Formulæ

**Published:** 1885-09-20

**Authors:** 


### PRACTICAL NOTES AND FORMULÆ.

**For Vomiting, Cholera, Etc.**—

Dr. J. W. Trader, of Missouri, writes—.

*Editor Southern Medical Record :*

As there seems to be some points not well understood, in my essay before the Missouri Medical Association, at our annual meeting in St. Joe, Mo., in May last, I submit the following as my formula for the "Cholera Mixture" :

| | | |
|---|---|---|
| ℞. | Glycorol of carbolic acid, . . . | aa . . . . . . . . . . . . . . . ℥ j. |
| | Glycorol of chloroform . . . . . | |
| | Tincture of opii . . . . . . . . . . . | |
| | Tincture of matico . . . . . . . . . | |
| | Aqua camphora . . . . . . . . . . | |
| | Aqua menth pip . . . . . . . . . . . | |
| | Aqua menth virid . . . . . . . . . . | M. |

Sig : Fifteen to twenty drops in hot water, every five to ten minutes until relief is obtained.

The formula for the glyceroles are as follows :

| | | |
|---|---|---|
| ℞. | Chloroform . . . . . . . . . . . . . . . . . . . . . . . . . . . . . . . . . | ℥ j, |
| | Glycerine . . . . . . . . . . . . . . . . . . . . . . . . . . . . . . . . . . | ℥ v. |

Put into a sixteen-ounce bottle and shake until a perfect mixture is obtained, which will require some time. There should be no subsidence of the chloroform when the mixture is perfect.

| | | |
|---|---|---|
| ℞. | Carbolic acid (Calvert's) . . . . . . . . . . . . . . . . . . . . . . . | ℥ j, |
| | Glycerine . . . . . . . . . . . . . . . . . . . . . . . . . . . . . . . . . . | ℥ vi. |

Mix as above for the glycerole of chloroform.

I want to say this much in addition, for the benefit of my professional brethren, especially those of the South, where gastric disturbances are probably more prevalent than in the North : That I have never failed to relieve a case of vomiting, depending upon faulty nutrition. I have had no experience with it in cases of reflex disturbance, as the vomiting of pregnancy, etc., but firmly believe that it will not only relieve but cure a majority of the cases depending upon the cholera microbe—cholera morbus—and will prove a great boon in epidemic cholera.

An yfurther information that I can give is at your disposal.

**Earache.**—It is affirmed that a few drops of a 4-per cent. solution of cocaine put into the external meatus will promptly relieve the pain of that exceedingly annoying affection, the earache.

**Application for Inflamed Surfaces.**—Dr. Anderson, of London, gives the following as the most valuable application for inflamed surfaces that he has ever tried:

| | | |
|---|---|---|
| ℞. | Bismuth oxidi | 100 grains |
| | Acidi oleici | 14 drachms |
| | Ceræ alb | 5 drachms |
| | Vaseline | 15 drachms |
| | Ol. rosæ | mj. |

M. —*Medical Compend.*

**For Burns.**—The following is recommended by Dr. Bond as a topical application for burns and for the lubrication of catheters:

| | | |
|---|---|---|
| ℞. | Acidi borici | 15 grains |
| | Glycerin | 1 drachm |
| | Ol. olivæ | 1 ounce. |

M. Sig. Apply.—*Med. Comp.*

**Salicylate of Potassium in Acute Rheumatism.**—Dr. E. L. Miller, in the Therapeutic Gazette, says that he has been using salicylate of potassium in cases of acute articular rheumatism with much satisfaction. In cases where the salicylate of sodium caused intense nausea and vomiting, the potassium salicylate was substituted with a disappearance of the gastric irritation and a marvelous improvement in the condition of the patient. In one case, after twenty-four hours' use of the potassium salts, the joint pain ceased, and the temperature fell from 104.2° to 99.62. He also says that it is of benefit in the fermentative diseases of the stomach. The formula he recommends is as follows:

| | | |
|---|---|---|
| ℞. | Acidi salicylatis, Potassii bicarb., aa | ʒv |
| | Aquæ | ℥ij |
| Solve et add: | | |
| | Tinct. nucis vomicæ | ʒij |
| | Spr. lav. co | ʒij |
| | Syr. simplicis q. s. ad | ℥iv. |

M. Sig. A teaspoonful every three hours, well diluted.

**Stimulating Anodyne Liniment.**—Dr. N. G. Willis, in *New England Medical Monthly*, says the following has proved itself very useful in my hands where a stimulating anodyne liniment is desired:

| | | |
|---|---|---|
| ℞. | Chloroform, Tict. aconite leaves, aa | ℥ss |
| | Oil of organum, Aquæ ammonia, aa | ʒij |
| | Tinct. hyosciamus, Tr. capsicum, aa | ℥ss |
| | Tr. sapo. co., Tr. arnica, aa | ʒv |

**Nocturnal Emissions.**—Dr. T. L. Lallersteat writes: I notice a query on the above subject in August number. I cannot say what that prescription will do, but here is one that I have never known to fail:

| | | |
|---|---|---|
| ℞. | Bromide potassium | grs. 6.40 |
| | Fl. ext. gelsemium | gtts. 2.50 |
| | Tinct. veratrum vir | gtts. 1.28 |
| | Water 2 s. to make | oj. M. |

Dose—a tablespoonful at 8, 4 and 12, and on going to bed take a glass two-thirds full of water.

A case of aggravated dyspepsia with constipation was, by Prof. Da Costa, given:

| | | |
|---|---|---|
| ℞. | Tinct. capsici | 1 drop |
| | Tinct. nucis vom | 8 drops |
| | Tinc. gent. comp | 1 drop |

M. Sig—Ter die. With gr. 1-5 aloin at bedtime, and avoid starchy diet.—*Peoria Med. Monthly.*

Prof. Bartholow says that for hæmoptysis "ipecacuhana is a remarkable physiological remedy." In a case at the clinic it was given in combination, as follows:

| | | |
|---|---|---|
| ℞. | Ext. ipecacc. fluidi,<br>Ext. ergot. fluid, | aa 5 drops |

M. Sig.—Tablespoonful as required.—*Peoria Med. Monthly.*

The following combination, Prof. Bartholow's, for asthamatic attacks, has been found very useful:

| | | |
|---|---|---|
| ℞. | Ext. grindeliæ fluid | ½ ℥ |
| | Ext. lobeliæ fluid | 2 dr. |
| | Ext. belladon, fluid | 1 dr. |
| | Potassii iodidi | 3 dr. |
| | Glycerini | 3 ℥ |

M. Sig.—Tablespoonful as required.—*Peoria Med. Monthly.*

Dr. Horace Dobell says the following formula is one of the most satisfactory he has ever tried in constipation:

| | |
|---|---|
| Podophyllin | 2 gr. |
| Essence of ginger | 2 dr. |
| Alcohol | 2 ℥ |

M. Sig: A teaspoonful at bedtime, in a wineglassful of water, nightly, or every second or third night.—*Ind. Med. Jour.*

**Antidote for Rhus Poisoning.**—Old sour butter-milk or lactic acid applied frequently to the parts affected will quickly antidote the rhus poisoning. Have used it frequently.—*W. D. Gentry in Medical Investigator.*

